# OVLT lesion decreases basal arterial pressure and the chronic hypertensive response to AngII in rats on a high-salt diet

**DOI:** 10.1002/phy2.128

**Published:** 2013-10-23

**Authors:** John P Collister, Marin K Olson, David B Nahey, Alexandre A Vieira, John W Osborn

**Affiliations:** 1 Department of Veterinary and Biomedical Sciences, College of Veterinary Medicine, University of MinnesotaSt. Paul, MN; 2 Department of Integrative Biology and Physiology, Medical School, University of MinnesotaMinneapolis, MN

**Keywords:** Angtiotensin II, circumventricular organ, hypertension, OVLT

## Abstract

We have reported that lesion of the organum vasculosum of the lamina terminalis (OVLT) has no effect on basal levels of mean arterial pressure (MAP) but abolishes the hypertensive effects of angiotensin II (AngII) in rats consuming a normal-salt diet. These results suggest that the OVLT does not contribute to regulation of MAP under conditions of normal salt intake, but it is an important brain site for the hypertensive actions of AngII. The OVLT has been proposed as a major sodium sensor in the brain and the hypertensive effects of AngII are exacerbated by high-salt intake. Therefore, the objective of this study was to investigate the role of the OVLT during AngII-induced hypertension in rats fed a high-salt diet. Male Sprague-Dawley rats underwent sham (Sham; *n* = 9) or OVLT lesion (OVLTx; *n* = 8) surgery and were placed on a high-salt (2% NaCl) diet. MAP was measured by radio telemetry during three control days, 10 days of AngII infusion (10 ng/kg/min, i.v.), and a 3-day recovery period. MAP was significantly lower in OVLTx (97 ± 2 mmHg) compared to Sham (106 ± 1 mmHg) rats during the control period (*P* < 0.05). Moreover, the chronic pressor response to AngII was markedly attenuated in OVLTx rats. MAP increased 58 ± 3 mmHg in Sham rats by Day 10 of AngII compared to a 40 ± 7 mmHg increase in OVLTx rats (*P* < 0.05). We conclude that (1) the OVLT regulates the basal levels of MAP in rats consuming a high-salt and (2) the OVLT is an important brain site of action for the pathogenesis of AngII-salt hypertension in the rat. Supported by HL076312.

## Introduction

Hypertension induced by administration of angiotensin II (AngII) is driven, in part, by activation of the sympathetic nervous system (Brody et al. 1978; Lappe and Brody 1984; Bruner and Fink 1986; Osborn et al. 2007). Additionally, it is now known that this sympathoexcitatory action of AngII is exacerbated by increased salt intake (Cowley and McCaa 1976; Hall 1986; Osborn et al. 2007; Osborn and Fink 2010). Therefore, it is important to make the distinction between the “AngII-induced model” (normal salt intake) and the “AngII-salt model” (high-salt intake) with the realization that the neurogenic component is more significant in the latter. The location in the brain for the converging signals of dietary salt and circulating AngII that increase sympathetic activity are not fully understood but most likely involve forebrain sites (Osborn et al. 2007).

For example, since they possess a weak blood–brain barrier (Johnson and Loewy 1990) and high numbers of AngII AT_1_ receptors (Allen et al. 2000), forebrain circumventricular organs (CVOs) such as the subfornical organ (SFO) and organum vasculosum of the lamina terminalis (OVLT) have been implicated in the central actions of circulating AngII in a number of studies (Mangiapane and Simpson 1980a,b; McKinley et al. 1998; Hendel and Collister 2005; Vieira et al. 2010). Historically, the entire anterior ventral portion of the third ventricle (AV3V) has been implicated in both sodium and water homeostasis (Sladek and Johnson 1983; Buggy and Brody 1977) as well as chronic blood pressure regulation (Brody et al. 1978) as its ablation prevents several forms of experimental neurogenic hypertension (Buggy et al. 1977; Brody et al. 1978). The OVLT, together with the ventral median preoptic nucleus (MnPO) of the hypothalamus and efferent fibers of SFO, comprise the major structures of the AV3V region of the brain.

Both the OVLT and SFO are responsive to AngII (Mangiapane and Simpson 1980a,b; McKinley et al. 1992, 1998), and project to the MnPO (Miselis 1981; McKinley et al. 1992). The MnPO is an established relay station with projections to the hypothalamic paraventricular nucleus (PVN) (Xu and Herbert 1995), which in turn drives sympathetic activity via excitatory input to sympathetic premotor neurons of the rostral ventrolateral medulla, as well as preganglionic sympathetic cell bodies of the spinal cord (Bains and Ferguson 1995; Stocker and Toney 2005). In recent studies, we investigated the individual contributions of the SFO, MnPO, and OVLT to the AngII-induced model (normal salt intake) of hypertension. Discrete lesions of each structure attenuated or nearly eliminated the hypertensive response to 10 days of AngII administration to rats consuming a normal-salt diet (Hendel and Collister 2005; Ployngam and Collister 2007, 2008; Vieira et al. 2010).

The mechanisms whereby increased salt intake amplifies the neurogenic component of AngII hypertension are not known, but forebrain CVOs have been proposed as sites of convergence for these two signals (Osborn et al. 2007; Osborn and Fink 2010). Although more than one site may be required, another possibility is that a single brain site, which is responsive to AngII, is also responsive to increased plasma sodium/osmolality. For example, PVN projecting neurons from the SFO can be activated by both AngII and hyperosmolality (Gutman et al. 1988; Anderson et al. 2000). Therefore, we recently tested the hypothesis that similar to what has been proposed for the murine model of AngII-induced hypertension (Zimmerman et al. 2004; Young et al. 2012) the SFO is the sole brain site responsible for AngII-salt hypertension in the rat. Contrary to our hypothesis, lesion of the SFO had minor effects on the arterial pressure response to AngII in this model (Osborn et al. 2011b). Moreover, the magnitude of the effect of SFO lesion on the arterial pressure response to AngII was similar to what we observed in rats on a normal-sodium diet (Hendel and Collister 2005). Therefore, we concluded the SFO is not responsible for the synergistic effects of AngII and a high-salt diet, and that other CVOs must be involved.

The OVLT has been implicated in mediating the sympathetic nerve activity response to acute increases in plasma osmolality, and has been proposed to act as a “central osmoreceptor” (Toney et al. 2003; Toney and Stocker 2010). More recently it has been shown that OVLT lesion prevents the enhanced acute sympathoexcitatory and arterial pressure responses to activation of rostral ventrolateral medulla (RVLM) sympathetic premotor neurons observed in rats consuming a high-salt diet (Adams et al. 2009). Based on these studies, and our recent report that OVLT lesion prevents AngII-induced hypertension in rats on a normal-salt diet (Vieira et al. 2010), we hypothesized that lesion of the OVLT would also attenuate AngII-salt hypertension. We tested this hypothesis by comparing the cardiovascular and fluid balance responses to 10 days of AngII administration between OVLT-lesioned and sham-operated rats consuming a high (2.0%)-NaCl diet. The novel findings of this study were as follows: in contrast to previous studies in which SFO lesion had minor effects on blood pressure in the AngII-salt model of hypertension (Osborn et al. 2011b), the current results showed that OVLT lesion significantly decreased basal levels of arterial pressure during high dietary salt, as well as the magnitude of the hypertensive response to AngII.

## Material and Methods

All methods were approved by the Institutional Animal Care and Use Committee and conducted in accordance with institutional and National Institutes of Health guidelines. Male Sprague-Dawley rats (Charles River Laboratory, Wilmington, MA) weighing 250–275 g were housed in an animal housing facility with a 12–12 h light–dark cycle and provided with fresh distilled water and high-salt diet (2.0% NaCl) rat pellets (Research Diets, New Brunswick, NJ) ad libitum until the beginning of the protocol. Following the OVLT lesion or Sham surgeries, rats were housed in metabolic cages (Nalgene; Nalge Nunc International, Rochester, NY) and provided distilled water and powdered high-salt diet (2.0% NaCl; Research Diets) ad libitum.

### Surgical procedures

Rats were randomly selected for either electrolytic lesion of the (OVLTx; *n* = 8) or sham operation (OVLTshm; *n* = 9) as previously described (Vieira et al. 2010). Rats were anesthetized with ketamine and atropine (75 mg/kg body wt i.p. and 0.2 mg/kg body wt i.p., respectively) and placed in a stereotaxic frame (model no. 900; David Kopf Instruments, Tujunga, CA). A dorsal midline incision was made in the skin of the skull. Bregma and lambda were exposed, repositioned to be on the same horizontal level, and a 2.0-mm hole was drilled into the skull. An 0.008 in. diameter Teflon-coated monopolar tungsten electrode, with 0.5 mm bared at the tip, was inserted into the brain using the following coordinates: +0.6 mm from bregma, on the midline, and −8.00 mm below the dura mater. Electrolytic lesions were performed using a cathode current (1.0 mA for 20 sec). Sham rats underwent the same surgical procedures, with the exception that ventral coordinates were 4.0 mm less so as not to damage the OVLT, and no current was passed. The hole in the skull was repaired with bone wax and the skin closed with 3–0 (0.2 mm) silk suture. After surgery, rats were given an injection of the antibiotic gentamicin (2.5 mg, i.m.) and the analgesic butorphanol tartrate (0.075 mg, s.c.).

One week after lesion or sham operation, rats were instrumented with radiotelemetric pressure transducers (model no. TA11PAC40; Data Sciences International, Inc., Saint Paul, MN) for continuous 24-h (500 Hz for 10 sec/min) sampling of mean arterial pressure (MAP) and heart rate (HR). Rats were anesthetized with pentobarbital (75 mg/kg i.p.), a 2.0-cm incision was made in the groin area and any visible connective tissue teased apart to visualize the left femoral artery and vein. Then, a 3.0-cm midline abdominal incision was made to expose the abdominal cavity and the transmitter catheter was passed through the abdominal muscles with a 15 gauge hypodermic needle. The body of the transmitter was secured into the abdominal cavity by suturing it to the abdominal muscles with 3–0 (0.2 mm) silk suture thread and the skin was closed with surgical staples. The femoral artery was isolated, a small incision made, and the catheter was advanced proximally into the femoral artery so the tip was located in the abdominal aorta. The catheter was tied to the vessel with 3–0 (0.2 mm) silk suture, and a drop of medical adhesive (Vetbound TM; 3M, Saint Paul, MN) was applied to secure the catheter in place. The abdominal muscle was closed with 3–0 (0.2 mm) silk suture and the skin closed with surgical staples.

A catheter was then implanted in the femoral vein for continuous venous infusion and acute injections. The catheter was tunneled subcutaneously, exteriorized between the scapula through a dacron mesh button tether (Harvard Apparatus Inc., Holliston, MA), and the mesh sutured to the interscapular muscle. The tether was connected to a spring, which was attached to a freely moving swivel. A single-channel hydraulic swivel was used to connect the catheter to an infusion pump. Following the telemetric instrumentation and catheter implantation, rats received an injection of the analgesic butorphanol tartrate (0.075 mg, s.c.). Each rat received daily prophylactic antibiotic ampicillin (15 mg, i.v.) for 3 days following instrumentation. Rats also received a continuous intravenous infusion of 0.9% sterile NaCl (7 mL/24 h).

### Experimental protocol

After 1 week of recovery, rats entered the following protocol: 3 days of baseline control, 10 days of intravenous AngII infusion (10 ng kg^−1^ min^−1^), and 3 days of recovery. AngII was dissolved in sterile 0.9% saline and given at a rate of 7 mL/24 h. During control and recovery periods, all rats received intravenous infusion of normal saline (7 mL/24 h).

The daily food and water intake and urine output were measured gravimetrically. Sodium intake was calculated as the sum of the sodium received from the daily intravenous infusion (1 mmol/day), plus the product of the food intake and the sodium content of the food (2.0% NaCl, 0.35 mmol/g). Urinary sodium concentration was measured with an ion-specific electrode (NOVA-5+; Biomedical, Waltham, MA). The daily urinary sodium excretion was calculated as the product of the urine output and urinary sodium concentration. The daily sodium and water balances were calculated as the difference between intake and urinary excretion of sodium and water, respectively.

At the end of the experiment, rats were deeply anesthetized with a lethal dose of pentobarbital sodium and perfused transcardially with 4% paraformaldehyde in phosphate-buffered saline. Brains were collected, frozen, cut coronally (50-μm sections), stained with cresyl violet, and analyzed by light microscopy to confirm the site of the OVLT lesion.

The results are reported as means ± SE, One- or two-way analysis of variance (ANOVA) combined with a Student–Newman–Keuls test was used for comparisons. Differences were considered significant at *P* < 0.05.

## Results

### Histology

**Figure 1:**
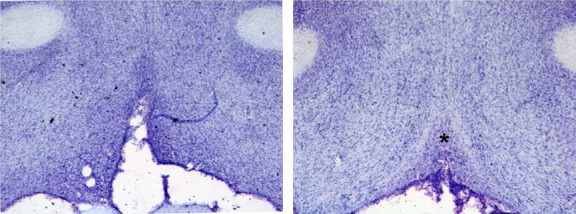
Photomicrographs of hypothalamic coronal sections from an organum vasculosum of the lamina terminalis (OVLT) (left) lesioned rat and from a sham (right)-lesioned rat showing an intact OVLT (*).

Nine of the 25 performed lesions were considered complete (≥90% ablation) with no or minimal damage to periventricular structures surrounding the OVLT and only these were included in the following analyses. Figure 1 demonstrates representative coronal forebrain sections of complete (left) OVLT lesion and of Sham (right) operation.

### Effect of OVLT lesion on AngII-salt hypertension

**Figure 2:**
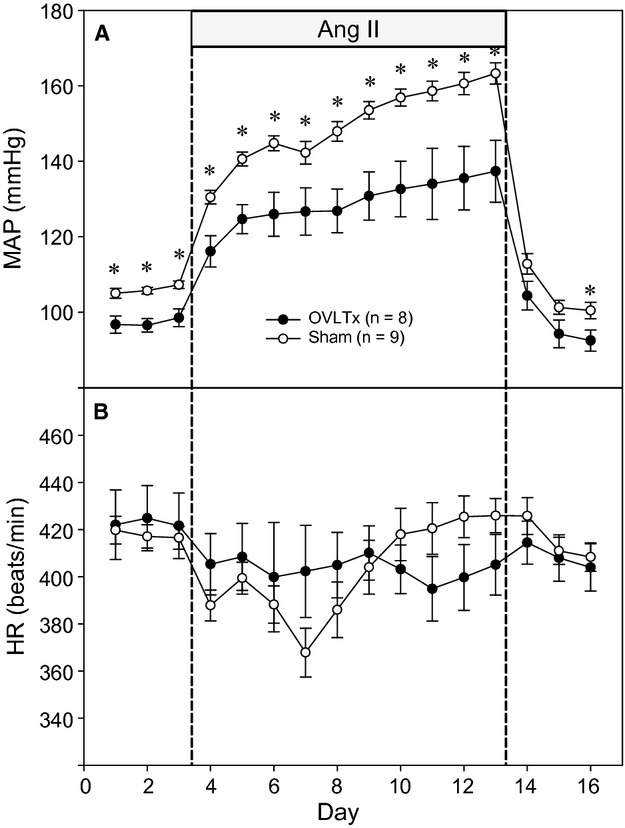
Average 24-h mean arterial pressure (A) and heart rate (B) recorded during saline infusion (3 days of control and recovery period) and 10 days of AngII infusion (10 ng kg^−1^ min^−1^) in organum vasculosum of the lamina terminalis (OVLT) and sham-lesioned rats. *P < 0.05 between lesioned and sham rats.

The response of (MAP; top panel) and (HR; bottom panel) to AngII infusion can be seen in Figure 2. OVLTx rats had statistically lower baseline MAP compared to Sham rats during the control period (average during 3 days: 97 ± 2 and 106 ± 1 mmHg, respectively). Additionally, MAP was higher in sham-operated rats throughout AngII treatment and during the recovery period. A statistical difference in MAP between groups was observed on all days during the experimental protocol, excluding the first 2 days of the recovery period (*P* < 0.05). HR was not different between OVLTx and Sham groups during the 3-day control period (average during 3 days: 423 ± 32 and 418 ± 15 beats/min, respectively), and no significance was reached throughout the experimental protocol.

**Figure 3:**
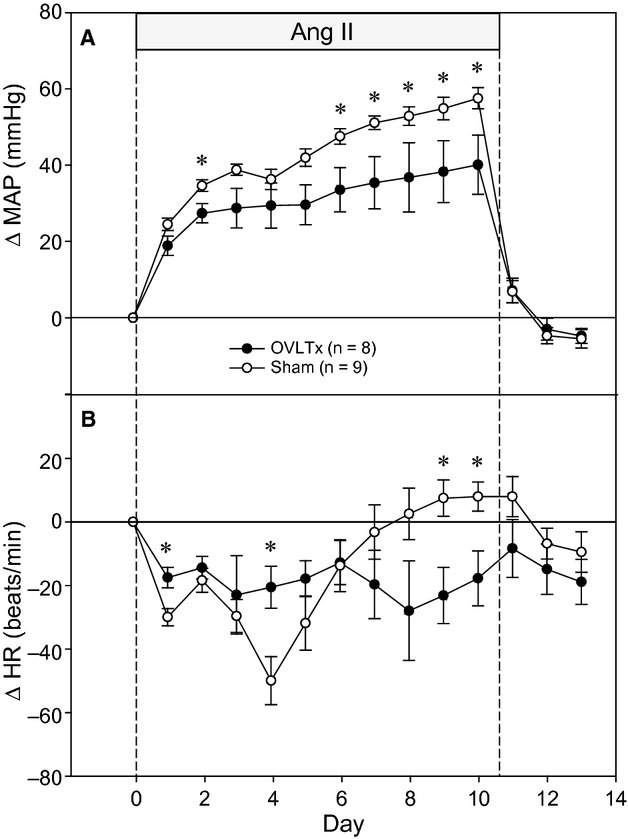
Change from baseline mean arterial pressure (A) and heart rate (B) recorded during saline infusion (3 days of recovery period) and 10 days of AngII infusion (10 ng kg^−1^ min^−1^) in organum vasculosum of the lamina terminalis (OVLT) and sham-lesioned rats. *P < 0.05 between lesioned and sham rats.

In order to compensate for the baseline differences in MAP between OVLTx and Sham rats, the changes in MAP (top panel) and HR (bottom panel) from baseline were calculated and are shown in Figure 3. The hypertensive response to AngII was significantly less in OVLTx compared to Sham rats on Day 2 and Days 4 through 10 (*P* < 0.05), having increased 58 ± 3 mmHg in sham rats and only 40 ± 7 mmHg in lesioned rats by Day 10 of AngII. HR in Sham rats initially decreased during the first 4 days of AngII but then rapidly increased nearly 60 beats/min from this point by Day 10 of AngII. The nadir of the HR response (Day 4) and subsequent increase coincided with a second phase of the hypertensive response as well (Fig. 3A). In contrast, the HR response was relatively flat in OVLTx rats (Fig. 3B) and the delta MAP did not exhibit a secondary increase after Day 4 (Fig. 3A).

### Effect of OVLT lesion on sodium and water balance

**Figure 4:**
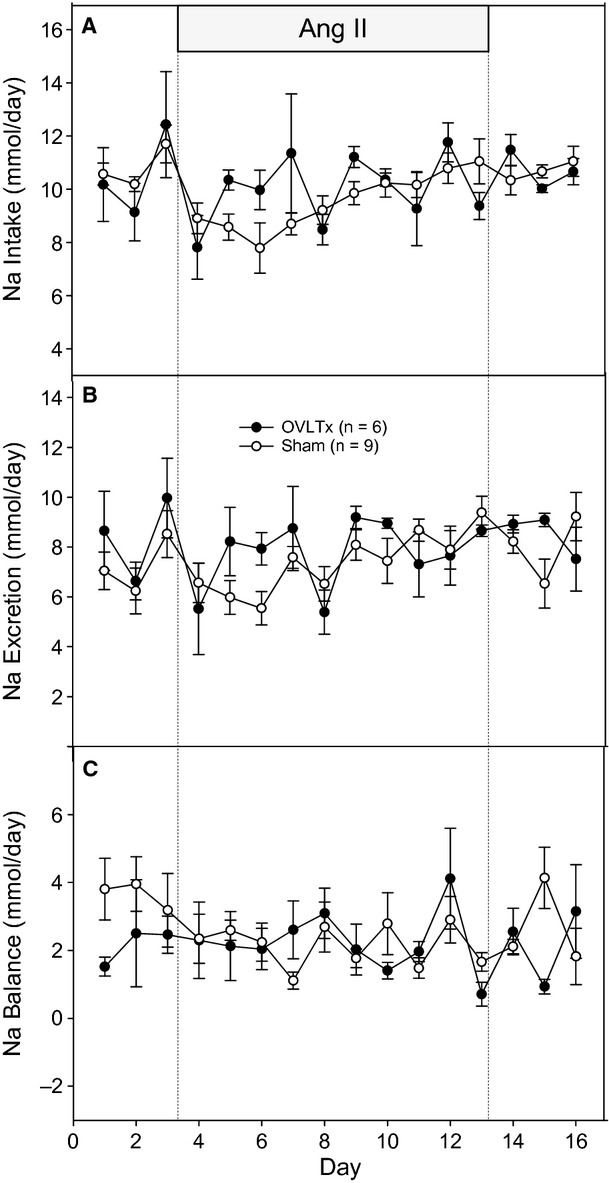
Average 24-h sodium intake (A) sodium output (B) and sodium balance (C) during saline infusion (3 days of control and recovery period) and 10 days of treatment in organum vasculosum of the lamina terminalis (OVLT) and sham-lesioned rats.

**Figure 5:**
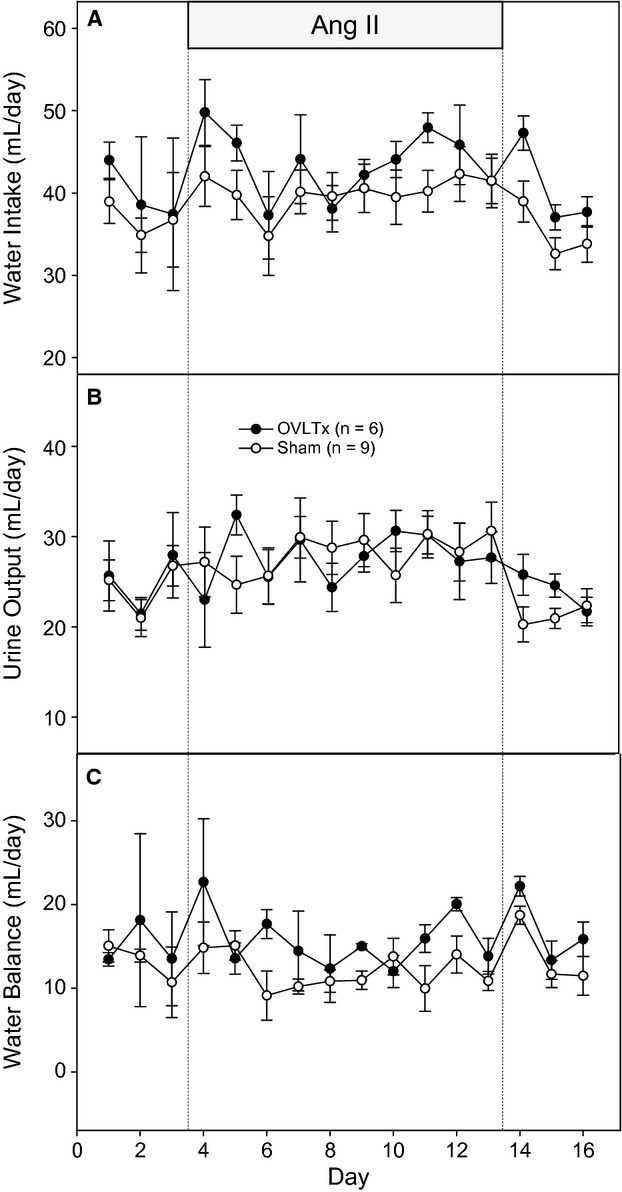
Average 24-h water intake (A) urine output (B) and water balance (C) during saline infusion (3 days of control and recovery period) and 10 days of treatment in organum vasculosum of the lamina terminalis (OVLT) and sham-lesioned rats.

Figure 4 shows sodium intake, sodium excretion, and sodium balance. These data show that there was no statistical significance in overall sodium balance between lesioned and sham-operated rats. Figure 5 displays water intake, urine output, and water balance. Similar to sodium balance, there was no statistical significance between groups during the entire protocol.

## Discussion

Because of their weak blood–brain barrier (Johnson and Loewy 1990), CVOs are responsive to acute and chronic changes in plasma osmolality and circulating hormones such as AngII. Previous studies from our laboratories have shown that lesion of the SFO or OVLT attenuates the hypertensive response to exogenously administered circulating AngII in rats with a *normal*-sodium intake (i.e., AngII-induced hypertension) (Hendel and Collister 2005; Vieira et al. 2010). However, since it is now known that the neurogenic component of the hypertensive response to AngII is directly related to salt intake (Cowley and McCaa 1976; Osborn et al. 2003; Osborn et al. 2007; Osborn and Fink 2010), we have further investigated the contribution of these CVOs to the pathogenesis of hypertension caused by AngII administration combined with *increased dietary salt* (i.e., the AngII-salt model). In a recent study, we showed that, contrary to our hypothesis, lesion of the SFO had minor effects on arterial pressure in AngII-salt rats (Osborn et al. 2011b). Therefore, the objective of this study was to investigate the role of the OVLT in AngII-salt model of hypertension in the rat. As such, we report three novel findings as discussed in the following sections.

### OVLT lesion decreases baseline arterial pressure in rats on a high-salt diet

In rats consuming a *high-salt* diet, those with lesion of the OVLT had lower basal levels of arterial pressure compared to the sham control group. This is in contrast to our recent study in which OVLTx had no effect on baseline MAP in rats on a *normal-salt* (0.4% NaCl) diet (Vieira et al. 2010). Moreover, we have also reported that lesion of the SFO has no effect on the basal level of arterial pressure in rats on either normal- (Hendel and Collister 2005) or high (Osborn et al. 2011b)-salt diets. To our knowledge, this effect of OVLTx has not been previously reported, nor have we observed this specific finding in studies performed with lesions of other CVOs or forebrain nuclei (area postrema or MnPO). This finding is consistent with a study showing that the OVLT is an important site for mediating the effects of chronic high-salt intake on increased excitability of sympathetic premotor neurons in the RVLM (Adams et al. 2009). Our findings support this view and suggest that this increased excitability of RVLM premotor neurons in rats on a high-salt diet results in a modest, but measureable, increase in arterial pressure that is dependent on an intact OVLT.

### OVLT lesion attenuates the hypertensive response to AngII in rats on a high-salt diet

The primary objective of this study was to determine the extent to which OVLTx prevents AngII-salt hypertension. We have previously shown that lesion of the OVLT prevents AngII-induced hypertension in rats on a normal-salt diet (Vieira et al. 2010). Similarly, in this study, OVLTx markedly attenuated the hypertensive response to AngII in rats consuming a high-salt diet. In Sham rats, MAP increased 58 ± 3 mmHg by Day 10 of AngII compared to 40 ± 7 mmHg in OVLTx rats. It is important to note that the steady-state MAP response to AngII in normal-salt rats (16 ± 4 mmHg) (Vieira et al. 2010) was much less than observed in this study, supporting the view that the sympathoexcitatory actions of AngII are directly related to salt intake. The magnitude of the effect of OVLTx on AngII-induced hypertension (i.e., the difference between OVLTx and Sham rats) was greater in high-salt (∼20 mmHg) than normal-salt (∼10 mmHg) rats (Vieira et al. 2010). Further studies are needed to firmly establish whether these differences are the result of synergistic or additive inputs of AngII and salt intake to central sympathetic pathways.

Finally, as illustrated by the present data, it is important to note that ability of OVLTx to attenuate AngII-induced hypertension in normal- and high-salt rats, is greater than what we have seen in similar SFO lesion studies (Hendel and Collister 2005; Osborn et al. 2011b). Taken as a whole then, studies from our laboratories suggest that the OVLT plays a more significant role in AngII-salt hypertension than the SFO. This is a significant contrast to studies in a murine model of AngII-induced hypertension, in which the SFO has been proposed to be the predominant forebrain site of action of AngII (Zimmerman et al. 2004; Young et al. 2012). It should be noted that a decreased sensitivity of MAP in response to AngII (with or without increased sodium) has been reported in mice compared to rats (Cholewa et al. 2005). However, the role of CVOs in modulating salt sensitivity of AngII-induced hypertension has not been studied in the mouse. Further studies are needed to establish whether these discrepant findings are due to species or methodological differences.

### OVLT lesion alters the temporal profile of arterial and HR responses to AngII administration in high-salt rats

Several published studies indicate that AngII-salt hypertension is associated with delayed activation of the sympathetic nervous system. This idea is based on measurements of whole body norepinephrine spillover (King et al. 2008), the acute depressor response to ganglionic blockade (Hendel and Collister 2005; Vieira et al. 2010), and recently published studies of neural regulation of HR and splanchnic vascular resistance in AngII-salt rats (Kuroki et al. 2012). All of these studies are consistent with the idea that AngII increases sympathetic activity in a delayed fashion, typically beginning ∼5 days after initiation of AngII administration, and only in rats on a high-salt diet.

This study further supports this notion along with new findings suggesting the OVLT is an important brain site for mediating these responses. For example, HR initially *decreased* ∼ 50 beats/min during the first 4 days of AngII in Sham rats on a high-salt diet (see Fig. 3B), a response that was most likely mediated by baroreceptor reflexes even though AngII does attenuate the baroreflex control of HR (Guo and Abboud 1984). HR then rapidly *increased* ∼60 beats/min from the nadir over the next 6 days, despite the fact that arterial pressure continued to rise. This response is consistent with the idea that, beginning on Day 5, the combination of AngII and high salt activates “non-baroreflex” pathways that increase HR. More importantly, as shown in Figure 3B, the rapid “switch” of HR control was absent in OVLTx rats. Finally, it is important to note that this temporal profile of HR in response to AngII is not seen in normal-salt rats and lesion of the OVLT has no effect on HR under normal-salt conditions (Vieira et al. 2010). Close examination of the AngII-induced changes in MAP (see Fig. 3A) in both groups shows a similar temporal pattern. Sham rats exhibited a biphasic arterial pressure response in which MAP rapidly increased during the first 4 days of AngII, followed by a slower phase beginning on Day 5. This is consistent with the early “non-neurogenic phase” following by a later “neurogenic phase” of AngII-salt hypertension as previously described (Osborn et al. 2011a). More importantly, the second slower phase of the MAP response was not observed in OVLTx rats. Indeed, we have reported this similar temporal phenomenon of attenuation of the hypertensive effects of AngII at approximately Day 5 of AngII treatment in other previous studies (Hendel and Collister 2005; Ployngam and Collister 2007, 2008; Osborn et al. 2011b).

### Summary

OVLT neurons have been proposed to act as “central osmoreceptors” owing to their role in mediating sympathetic nerve responses to increased plasma osmolality (Toney and Stocker 2010; Toney et al. 2003). The OVLT targets downstream neurons of the PVN, which send excitatory efferent projections the RVLM (Adams et al. 2009). In recent studies, it has been shown that rats on a high-salt diet have an increased sympathoexcitatory response to activation of sympathetic premotor neurons in the RVLM, and this response is abolished by lesion of the OVLT (Adams et al. 2009). This is consistent with our observation that lesion of the OVLT decreases basal levels of arterial pressure in rats consuming a high-salt diet. Finally, we conclude that the delayed neurogenic phase of hypertension-induced by AngII administration, in combination with a high-salt diet, is mediated in part by the OVLT.

### Perspectives

The data of this study support the idea that the OVLT is a major site in the forebrain for the synergistic actions of circulating AngII and increased salt intake. However, OVLT lesion did not prevent AngII-salt hypertension entirely. One possibility is that the delayed “neurogenic phase” of AngII-salt hypertension is also mediated, in part, by nonneurogenic mechanisms (i.e., salt and water retention). We do not believe this is likely since we have not observed altered water and or sodium balance during this phase of hypertension in of our studies. Another possibility is that no single site in the AV3V region is solely responsible for mediating neurogenic hypertension in the AngII-salt model. A third possibility is that other CVOs, such as the SFO, assume a greater role in the pathogenesis of AngII-salt hypertension in OVLTx animals. Indeed, there is some controversy as to the level of AT_1_ receptor expression in the SFO after chronic exposure to AngII (Wei et al. 2009; Nunes and Braga 2011).

These are important and currently unresolved issues that arise with lesion studies of this nature. The major objective of our studies has been to resolve the individual contribution of key regions of the AV3V to AngII-induced hypertension under conditions of normal- and high-salt intake. Taken as a whole our studies clearly show that, contrary to the view that the SFO is solely the key region of the AV3V responsible for mediating AngII-induced hypertension, the OVLT plays a predominant role. However, we conclude that no single site (SFO, MnPO, or OVLT) in the forebrain is responsible for AngII-salt hypertension.
